# Toxicity of Transition Metal Oxide Nanoparticles: Recent Insights from *in vitro* Studies

**DOI:** 10.3390/ma3104842

**Published:** 2010-10-25

**Authors:** Yue-Wern Huang, Chi-heng Wu, Robert S. Aronstam

**Affiliations:** Department of Biological Sciences and the Missouri S&T cDNA Resource Center, Missouri University of Science and Technology, 400 W. 11th Street, 105 Schrenk Hall, Rolla, MO 65409, USA; E-Mails: cwc33@mst.edu (C.-H.W.); aronstam@mst.edu (R.S.A.)

**Keywords:** nanoparticle, toxicity, metal oxide, oxidative stress, calcium homeostasis, signal transduction

## Abstract

Nanotechnology has evolved to play a prominent role in our economy. Increased use of nanomaterials poses potential human health risk. It is therefore critical to understand the nature and origin of the toxicity imposed by nanomaterials (nanotoxicity). In this article we review the toxicity of the transition metal oxides in the 4th period that are widely used in industry and biotechnology. Nanoparticle toxicity is compellingly related to oxidative stress and alteration of calcium homeostasis, gene expression, pro-inflammatory responses, and cellular signaling events. The precise physicochemical properties that dictate the toxicity of nanoparticles have yet to be defined, but may include element-specific surface catalytic activity (e.g., metallic, semiconducting properties), nanoparticle uptake, or nanoparticle dissolution. These *in vitro* studies substantially advance our understanding in mechanisms of toxicity, which may lead to safer design of nanomaterials.

## 1. Overview

Nanotechnology involves the study of the control of matter on atomic and molecular scales. Nanomaterials have at least one dimension in the range of 1–100 nm [1]. Nanotechnology is being applied in diverse fields, including extensions of conventional device physics, new approaches based upon molecular self-assembly, the development of novel materials with dimensions on the nanoscale, and even the direct control of matter on the atomic scale. The application of nanotechnology in biology (nanobiotechnology) encompasses development of nanomaterials for delivering and monitoring biologically active molecules, disease staging, therapeutical planning, surgical guidance, neuro-electronic interfaces, and electronic biosensors. 

In 2000 the U.S. National Science Foundation estimated that the market for nanotechnology products will be over one trillion US dollars by 2015. This increase in use will likely lead to unintended exposures to nanomaterials by occupational workers and end product users via inhalation, dermal absorption, or gastrointestinal tract absorption. In particular, the direct use of nanomaterials in humans for medical and cosmetic purposes dictates vigorous safety assessment of toxicity. Presently, the adverse effects of such exposure on human health and the environment are incompletely understood [2].

**Figure 1:**
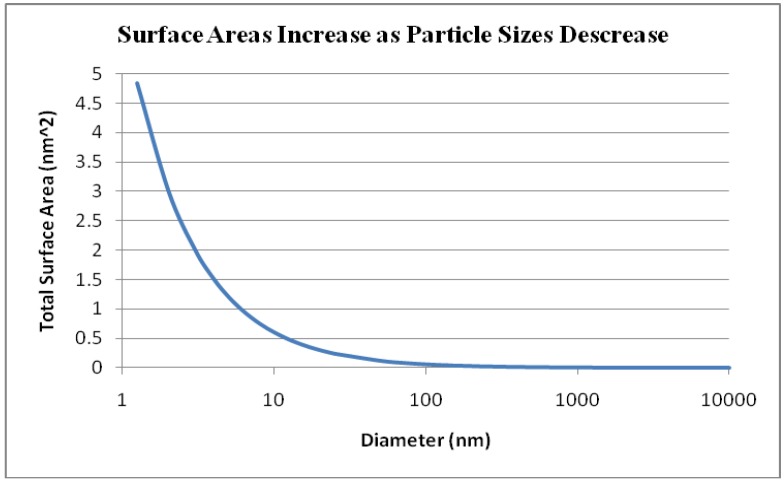
Assuming an equal particle mass, as particle sizes decrease, surface areas increase. (Yue-wern Huang and Hannah Huang, unpublished data).

Nanotoxicology is an emerging field that builds upon previous work on airborne particle toxicity. Given (1) fixed particle mass, (2) unitary density, and (3) particle surface bioreactivity, nanoparticles possess better tissue penetration and higher biological potency than coarse (2.5–10 µm) and fine (<2.5 µm) particles, reflecting their small sizes and large reactive surfaces (**Figure 1**). Inhaled particles and fibers generate oxidants including reactive oxygen species (ROS) and reactive nitrogen species (RNS) [3,4,5]. Knaapen *et al.* characterized the generation of cellular ROS/RNS as “primary source” if arising from the target cells themselves or “secondary source” if arising from inflammatory cells [6]. Oxidative stress (OS) plays important roles in cellular signaling, inflammatory, and genotoxic and proliferative responses [5,6,7,8,9,10,11]. A three-tiered response to oxidative stress model has been outlined [12]: The first tier involves the induction of antioxidant enzymes such as HO-1, NQO1, superoxide dismutase, catalases and glutathione peroxidases. If tier 1 protection fails to restore cellular redox equilibrium, tier 2 responses involving activation of pro-inflammatory signaling pathways such as the JNK and NF-κB cascades are triggered. At higher and more prolonged oxidative stress levels, cellular perturbation and disarray result in a decrease in mitochondrial membrane potential, leading to cell death.

## 2. Transition Metal Oxide Nanoparticles and their Applications

Transition metal oxides are used in catalysis [13], magnetocooling [14], optical and recording devices [15,16], purification of enzymes and other biological materials [17], water purification devices [18], magnetic field assisted radionuclide therapy [19], embolics [20,21,22], and targeted drug delivery [23]. Among the transition metal oxides, titanium dioxide (TiO_2_), cupric oxide (CuO), and zinc oxide (ZnO) have received the most attention due to their unique physical and chemical properties.

TiO_2_ allows osseointegration of artificial medical implants and bone. TiO_2_ is also extensively used as a pigment, a thickener, and a UV absorber in cosmetic and skin care products. TiO_2_, particularly in the anatase form, is a photocatalyst under ultraviolet light. It can be employed for solar energy conversion: Dye, polymer, or quantum dot sensitized nanocrystalline TiO_2_ solar cells can be manufactured using conjugated polymers as solid electrolytes. TiO_2_ is also used as a material in the memristor, a new electronic circuit element.

Nanostructures of ZnO, including particles, rods, wires, belts, tubes, cages, walls, and rings, have found utility due to their unique nanoscaled electronic and optoelectronic properties [24,25,26,27,28,29,30]. ZnO has wide direct band gap (3.37 eV or 375 nm at room temperature), making it suitable for use in laser diodes and light emitting diodes. ZnO has high biocompatibility and fast electron transfer kinetics; these features enable the use of this material as a biomimic membrane to immobilize and modify biomolecules [31]. Other applications are in catalysis, paints, abrasives, wave filters, UV detectors, transparent conductive films, varistors, gas sensing, solar cells, sunscreens, and cosmetic products.

Cupric oxide (CuO) is used as a pigment in ceramics to produce blue, red, and green (and sometimes gray, pink, or black) glazes. CuO is a p-type semiconductor due to its narrow band gap of 1.2 eV. CuO can be used to produce dry cell batteries as well as wet cell batteries as the cathode. CuO is used as an abrasive to polish optical equipment, and it can be used to dispose of hazardous materials such as cyanide, hydrocarbons, halogenated hydrocarbons and dioxins via oxidation processes.

## 3. Methodologies to Study Toxicity of Transition Metal Oxides

Both *in vitro* and *in vivo* methods provide valuable tools to investigate nanotoxicity. In this article, we review *in vitro* studies. *In vitro* toxicity testing is a cost effective tool to evaluate the toxicity of nanomaterials; there are too many nanomaterials to evaluate each *in vivo*. Some advantages of *in vitro* studies using various cell lines are that they: (1) reveal effects of target cells in the absence of secondary effects caused by inflammation; (2) permit the identification of primary mechanisms of toxicity in the absence of the physiological and compensatory factors that confound the interpretation of whole animal studies; and (3) are efficient, rapid and cost-effective; and 4) can be used to improve design of subsequent expensive whole animal studies. For instance, we and others have used A549 (human bronchoalveolar carcinoma-derived cells), U87 (astrocytoma cells), U937 (human monoblastoid cells), mouse Leydig TM3 cells, human V79 and L929 fibroblasts, human SCCVII, B16F10 and FsaR tumor cells, and RAW264.7 (mouse peritoneal macrophage) cell lines to characterize oxidative stress-related signaling pathways [32,33,34,35,36,37,38,39,40,41,42]. A nontransformed human lung cell line (BEAS-2B) has been used to decipher expression of alteration of genes pertaining to oxidative stress and cell death pathways [43,44]. Co-culture of cell lines is another valuable technique to manipulate environmental influences *in vitro* studies [45].

A limitation of *in vitro* testing is that cells in culture do not experience the range of pathogenic effects observed *in vivo*, partly related to issues of translocation, toxicokinetics and coordinated tissue responses. A detail discussion of the limitations of *in vitro* studies can be found in Donaldson *et al.* [46].

## 4. Mechanisms of Cellular Uptake and Intracellular Interactions

Transmission electron microscopy (TEM) and scanning electron microscopy (SEM) have been the most widely used techniques to visualize agglomerated nanoparticles in cells [35,40,41,47]. To understand mechanisms of cellular uptake of nanoparticles, we recommend live cell imaging techniques, as cell fixation process can alter membrane properties leading to artificial uptake of nanoparticles [48]. Fluorescent probes such as Texas Red and FITC can be conjugated to nanoparticles to ascertain bioavailability. Studies on uptake mechanisms of transition metal oxide nanoparticles are limited; however, the following findings on the uptake of quantum dots, carbon nanotubes, gold nanoparticles, and silicon nanoparticles are likely to be relevant to transition metal oxides.

Cationic nanoparticles may enter cells more easily than anionic nanoparticles since cationic nanoparticles can interact with heparin sulfate proteoglycans on the membrane surface [49,50]. This increased bioavailability may explain why cationic silicon nanoparticles are more toxic than neutral and anionic silicon nanoparticles [51]. This electrostatic interaction is a prelude for a subsequent endocytic process. Exogenous materials can be internalized via multiple pathways, including clathrin-mediated endocytosis, caveolar-mediated endocytosis, macropinocytosis, phagocytosis, flotillin-dependent endocytosis, arf6-dependent endocytosis, IL2Rβ endocytic pathway, CLIC/GEEC endocytic pathway, circular doral ruffles, and trans-endocytosis. A few studies have suggested that the uptake of nanomaterials involves energy-, lipid raft- and actin-dependent macropinocytosis. Phagocytosis is an alternate proposed venue of entry. For instance, single wall carbon nanotubes conjugated with proteins as cargoes enter cells via energy-dependent pathways and become entrapped inside endosome/lysosomes [52]. We have observed that CdSe/ZnS quantum dots non-covalently conjugated with nona-arginines are internalized via lipid raft dependent macropinocytosis, and the complex is confined in lysosomes [53,54]. One study has demonstrated that Tat peptide-conjugated quantum dots (Tat-QD) are internalized via actin-dependent endocytosis, transported by microtubules, and aggregated around microtubule-organizing center; the Tat-QD vesicle are shed from filopodia [55]. On the other hand, gold nanoparticles conjugated with biomolecules are internalized via phagocytosis by mouse macrophages [56], and organic monolayer-coated silicon nanoparticles are phagocytosed by rat alveolar macrophage cells (NR8383) [51]. Clearly, studies on uptake mechanisms of transition metal oxides are needed.

## 5. Toxicity of Transition Metal Oxide Particles

Using commercially available raw nanoparticles to test toxicity is essential to understand human health risk and environmental impact, as this addresses unintended exposure. It is equally important to test surface modified-nanoparticles, as these modified nanoparticles have different kinetics and bioavailability than the native nanoparticles, and they are frequently applied directly on human body for purposes such as disease diagnostics, treatment, and prognostics.

**Figure 2:**
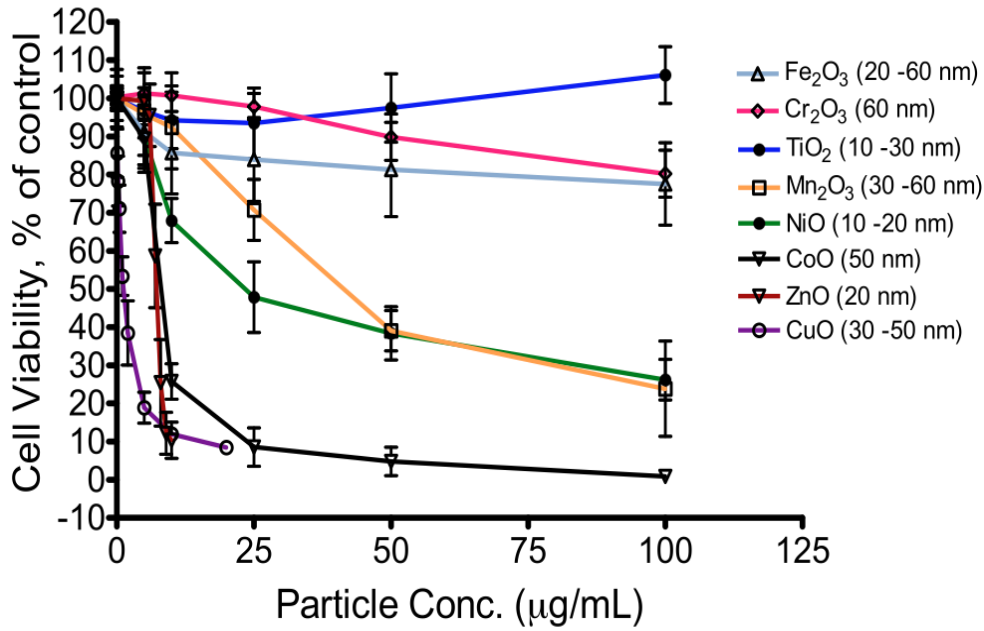
Cytotoxicity of eight transition metal oxides in BEAS-2B cells exposed for 24 h. (adapted from [43], with additional data).

The toxicological literature reveals a trend among these transition metal oxides: TiO_2_ is less toxic than CuO and ZnO in human cell lines [33,35,43,57,58]. A similar trend was observed in *E. Coli* [59] and yeast [60]. Using BEAS-2B cells, we investigated the toxicity of oxides of Cr, Mn, Fe, Co, Ni, Cu, and Zn, each of which is widely used in industry and is in the same period as Ti (V_2_O_3_ particles were excluded from this analysis because they are not commercially available in the same size range and morphology as the other compounds). Toxicity increased with atomic number (**Figure 2**), with the exceptions of Fe_2_O_3_ (lower toxicity than expected) and CoO (higher toxicity than expected). Fahmy and Cormier also identified a similar relationship of CuO and Fe_2_O_3_ toxicity in airway epithelial cells (HEp-2) [39]. It is difficult to compare the toxicity of TiO_2_ and V_2_O_5_, as synthesis methods, particle characteristics, and cell lines (Leydig TM3 cells, human V79 and L929 fibroblasts, human SCCVII, B16F10 and FsaR tumor cells) differ among the few published studies; however, it is quite clear that TiO_2_ is minimally toxic while V_2_O_5_ is toxic [40,41,42]. Data from these studies are consistent with our findings presented in Figure 2.

While the factors underlying this trend have not been established, several physicochemical properties have been considered, including element-specific catalytic activity on the particle surface, released ions (particle dissolution), and differential cellular uptake. In a study with A549 cells, Limbach *et al.* [61] demonstrated that 1) among titanium-, manganese-, iron-, or cobalt-doped silica, the addition of Ti or Fe has a much smaller effect on ROS generation than Mn due in part to surface catalytic activity; and 2) particles of Co_3_O_4_ and Mn_3_O_4_, but not TiO_2_ and Fe_2_O_3_, can significantly dissolve in a cell-free culture system leading to elevated ROS levels. They further demonstrated that much less ROS is formed when an aqueous iron solution contains A549 cells than when the solution contains no cells. The authors attributed this difference to a barrier function of the cell membrane for ions. It is also likely that that adsorption and binding of the metal oxide particles to the matrix of the cell medium may also be involved [62].

The potential role of dissolved ions in toxicity was also highlighted in a study by George *et al.* [63]. They found remarkably high levels of Zn^2+^ released into aqueous solutions from pure and Fe-doped ZnO nanoparticles. In one study, we suspended transition metal oxides in an aqueous solution for a period of 6 hours, and utilized a dialysis membrane with a molecular weight cutoff 500–1,000 to separate ionic species. We observed a trend of increased ion dissolution of metal oxides from TiO_2_ (0.0017%–0.0065%, wt based) to ZnO (1.25%–1.67%, wt based) (Huang *et al.* unpublished data), corresponding to the toxicity trend (Figure 2). While initial attempts to measure ion dissolution from metal oxide nanoparticles have been made by us and others, it would be desirable to assess ion dissolution in a cell medium relevant setting using a standardized protocol to separate dissolved ions. Moreover, pH maintenance during the experimental protocol is essential to allow comparisons with the cellular environment, and adsorption effects by culture medium can complicate the subsequent ICP-MS analysis and must be taken into consideration.

While Limbach’s findings [61] may explain the relatively low toxicity of Fe_2_O_3_, it is not obvious why CoO is more toxic than NiO. We suspect involvement of a variety of factors: surface catalytic activity, released ion, cell membrane as a barrier, adsorption to the matrix of the cell medium, cellular uptake, and possibly point of zero charge. Experiments using oxides of different oxidation states (CoO, Co_3_O_4_, NiO, Ni_2_O_3_) may be revealing in this regard.

Although we and others have contributed to the understanding of the physico-chemical properties governing cytotoxicity of transition metal oxides, there are still gaps in our knowledge. While studies on nanoparticles of industrial origin have contributed to risk assessment and management, these materials possess considerable heterogeneity in size, morphology, surface defects, *etc*. This makes it difficult to relate toxicity to specific particle characteristics. Thus, it is desirable to synthesize highly defined and uniform nanoparticles to identify the toxicity-contributing properties of nanoparticles for comparative and predictive nanotoxicology. Not only would this type of particles enrich our knowledge of particle physics, chemistry and biology, it would also assist in the design and production of safer alternative particles.

## 6. Mechanisms of Action of Toxicity

### 6.1. Exposure to Metal Oxides Tips Off Cellular Redox State—Elevated Oxidative Stress

**Figure 3:**
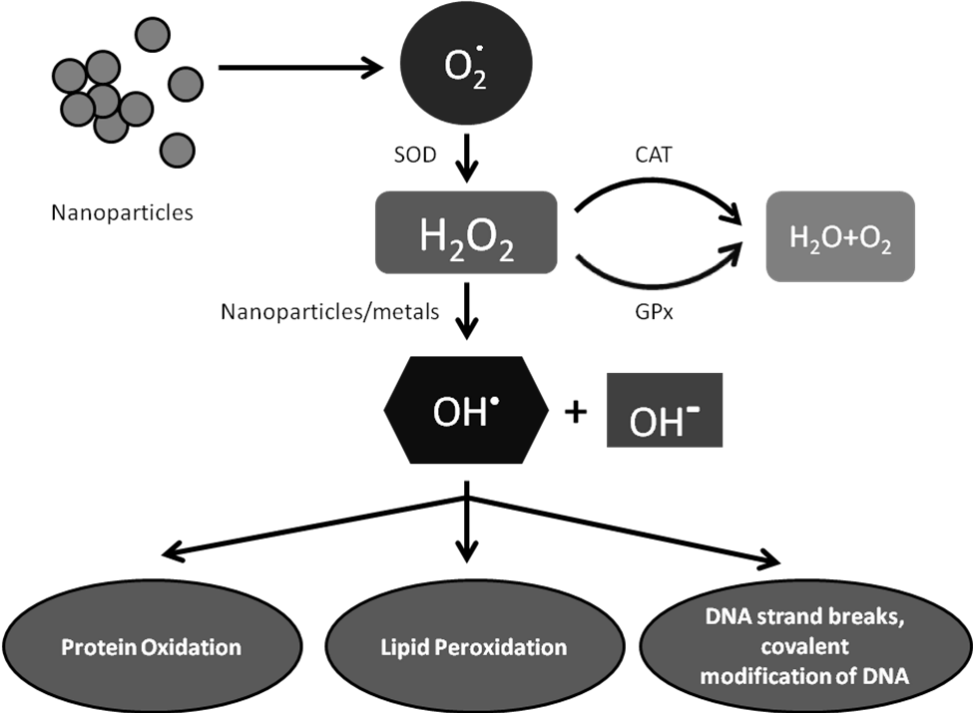
An example of metabolism of nanoparticle-induced oxidative stress and resulting toxicity.

Oxidative stress is a normal cellular process involved in many aspects of cellular signaling, though excessive oxidative stress can be harmful. Many studies have shown that exposure to nanoparticles elevates cellular oxidative stress [34,35,37,38,39,61]. To cope with elevated oxidative stress, cells mount protective or injurious responses. For instance, cells activate enzymatic and non-enzymatic antioxidant defense mechanisms. Glutathione peroxidases, catalases, superoxide dismutases, and phase II enzymes play essential roles in returning cells to a normal redox state (**Figure 3**). The transcription factor Nrf-2 has been shown to play an essential role in the antioxidant response element (ARE)-mediated expression of phase II detoxifying and antioxidant enzymes, as well as other stress-inducible genes, in response to oxidative stress [64,65,66]. A recent study with CeO_2_ nanoparticles has demonstrated that Nrf-2 can be activated and translocated into nucleus with subsequent induction of heme oxygenase-1 (HO-1) in CeO_2_-exposed BEAS-2B cells [67]. Nrf-2 can therefore be considered as a master switch for antioxidation defense, serving as a functional indicator of oxidative insult caused by nanomaterials. 

In our pathway-specific microarray study involving 84 oxidative stress responsive genes [20], exposure of BEAS-2B cells to a ROS-elevated but sublethal concentration of ZnO nanoparticles elevates the expression of genes pertaining to oxidative stress (PRDX3, PRNP, and TXNRD1 genes) and apoptosis (a pro-apoptotic BNIP3 gene). This is consistent with our biochemical and cytotoxicity findings. One caution in assessing gene regulation under oxidative stress is that the response is quite dynamic and dependent on somewhat arbitrary cutoff criteria relative to control levels. For instance, we did not find elevated expressions of several common antioxidant genes, including catalase, glutathione peroxidase, glutathione reductase, glutathione transferase, and superoxide dismutase in cells exposed to ZnO at the ROS-elevated but sublethal level [20]. This finding is consistent with results reported by Sarkar *et al.* [44] with toxic single-walled carbon nanotubes in BJ Foreskin cells. At different time points or concentrations (*i.e*., stress levels), gene expression may be alternately up-regulated or down-regulated.

Understanding the regulation of enzymatic and non-enzymatic antioxidant defense mechanisms may suggest strategies to mitigate elevated oxidative stress. For example, Gilliland *et al.* used knockout and genetic polymorphisms of genes that encode phase II enzymes to characterize a susceptibility mechanism that may explain why only some people develop PM-induced injury [68]. Catalase and glutathione peroxidase are two other examples: Catalase has a relatively low constitutive level in cardiomyocytes which predisposes cardiac muscle to oxidative stress damage. Cardiac muscle is also very susceptible to oxidative damage due in part to the rapid inactivation of glutathione peroxidase [69]. Overexpression of glutathione peroxidase in endothelial cells and myocytes significantly decreases oxidative stress-induced NF-kB activation, which leads to apoptosis [70]. Thus, tissue-specific responses to nanoparticle exposure should be considered when risk assessment is performed.

The fundamental factor that governs initiation of cellular oxidative stress by transition metal oxides is still unknown. Oxygen vacancies on metal-oxide surfaces are electrically and chemically active. Once entering circulatory systems or cells, charge accepting molecules, such as NO_2_ and O_2_, may be in close proximity at the vacancy sites; de-coupling of charged NO_2_ or O_2_ is possible and could be sources of initiation of oxidative stress. Interestingly, non-metal nanomaterials such as carbon nanotubes behave like (transition) metal oxides in inducing cellular oxidative stress. We hypothesize that oxidative stress initiated by metal or non-metal nanoparticles may be related to their metallic or semiconducting properties at nano-scale sizes. It remains to be determined how the metallic or semiconducting behaviors and metal dissolution differentially contribute to initiation of cellular oxidative stress and subsequent pathologic outcome.

When oxidative stress overwhelms defense mechanisms, cellular macromolecules such as proteins, lipids, and DNA are subject to damage. DNA damages include deletions, mutations, single- and double-strand breakages, adduct formation, and cross-linking with proteins. Studies have confirmed DNA adducts and oxidation-induced DNA fragmentation following exposure to metal oxide nanoparticles [35,58,71,72,73]. In response to DNA insult, cells attempt to repair the damaged DNA. Repair failure may lead to cell death (e.g., apoptosis) or cell transformation. It is important to identify types of DNA damages and repair mechanisms involved in nanotoxicity (e.g., nucleotide excision repair, base excision repair, mismatch repair, double strand repair, direct reversal), as this understanding may suggest measures for prevention or intervention, such as the over-expression of DNA repair genes.

In the case of severe damage to DNA, cells may die by either necrosis or apoptosis. Apoptosis is a complex and highly regulated process that is invoked to eliminate irreparably damaged cells. In contrast, necrosis has long been thought of as a disorderly event (although new evidence suggests otherwise), and is considered to be a consequence of extreme physiochemical stress. Although studies have shown that exposure to certain metal oxide nanoparticles induces apoptosis [74], it is unclear which pathways are involved. There are at least four major apoptotic pathways: extrinsic apoptotic (receptor mediated pathway), intrinsic apoptotic (mitochondrial pathway), caspase-2 dependent, and caspase-independent. Again, an understanding of which pathway(s) is involved in nanoparticle induced cell death may suggest measures for prevention or mitigation.

Insofar as multiple genes have been demonstrated to be involved in responses to oxidative stress, DNA damage, and cell death (apoptosis and necrosis), a custom, pathway-specific genetic approach could serve to decipher toxicity mechanisms of nanoparticles. On the other hand, whole genome approaches may serve as a discovery tool to identify unanticipated signaling pathways. 

### 6.2. Oxidative Stress and Perturbation of Intracellular Calcium Homeostasis

The intracellular calcium concentration ([Ca^2+^]_in_) plays major regulatory roles in cellular metabolism, signal transduction, and gene expression. For instance, calcium can influence cell cycle by activating protein kinase C (PKC), Ca^2+^/calmodulin-dependent protein kinases (CaMK), and mitogen-activated protein kinase (MAPK). Accordingly, [Ca^2+^]_in_ is tightly regulated, and alterations of [Ca^2+^]_in_ are associated with cellular dysfunction, metabolic and energetic imbalance, disease states, and cell death. A host of environmental toxicants elevate [Ca^2+^]_in_ directly or indirectly by promoting Ca^2+^ influx, releasing Ca^2+^ from intracellular stores, inhibiting Ca^2+^ sequestration, or blocking Ca^2+^ efflux from the cell [75]. A study with ultrafine particles demonstrated that calmodulin-dependent signaling pathways are crucial for cytotoxicity and cytoskeletal dysfunctions [76]. In our studies, ZnO caused a concentration-dependent elevation of [Ca^2+^]_in_, that could be partially attenuated by the antioxidant N-acetylcysteine (NAC), indicating an effect of oxidative stress on calcium homeostasis [43]. The inverse correlation between [Ca^2+^]_in_ and cell viability suggests a role for calcium in cell death. The moderation of this increase by nifedipine suggests that a portion of this increase reflects the influx of extracellular calcium. Membrane disruption (e.g., oxidative stress-induced lipid peroxidation) may also play a role in this influx. The involvement of Ca^2+^ release from intracellular stores has yet to be evaluated.

**Figure 4:**
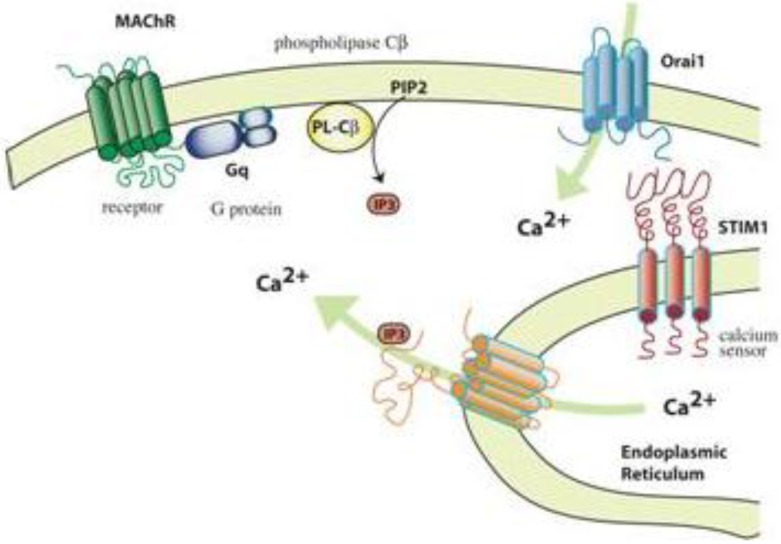
Store-operated Ca^2+^ entry (SOCE). Ligand binding to certain G protein coupled receptors leads to activation of phospholipase Cβ. The IP3 thus released increases Ca^2+^ release from the lumen of the endoplasmic reticulum. Depletion of ER calcium leads to a Stim1 (Ca^2+^ sensor)—Orai (Ca^2+^ channel) interaction and the entry of extracellular Ca^2+^. ZnO nanoparticles inhibit this pathway by blocking the SOCE without affecting proximal receptor signaling events [67].

In another study, the effects of ZnO nanoparticles on store operated calcium entry was studied in Chinese hamster ovary (CHO) cells stably transfected with a M3 muscarinic receptor. M3 receptors activate Gα_q_ transducer proteins that stimulate phospholipase Cβ (PLCβ) [77]. PLCβ hydrolyzes phosphatidylinositol 4.5-bisphosphate, releasing two second messengers, diacylglycerol and inositol trisphosphate (IP3). IP3 binds to the IP3 receptor on the endoplasmic reticulum (ER) to release calcium into the cytosol. Sensors in STIM1 proteins that are components of ER membranes detect the depletion of calcium in the ER. STIM1 proteins thus activated interact with Orai channel proteins in the plasma membrane to stimulate the entry of extracellular calcium, *i.e*., the store operated calcium entry (SOCE) (**Figure 4**) [78,79]. At a non-cytotoxic concentration (10 µg/mL), ZnO increased resting [Ca^2+^]_in_ of Chinese hamster ovary (CHO) cells expressing M3 muscarinic receptors from 40 to 130 nm without compromising calcium homeostatic mechanisms and the CHO cells had no store operated calcium entry response in the presence of 10 µg/ml ZnO. Hence, ZnO particles had minimal effects on IP3- or thapsigargin-mediated release of intracellular calcium from the endoplasmic reticulum, but strongly inhibited store operated calcium entry. This effect was seen a decrease in Ca^2+^ entry upon introduction of calcium to the extracellular medium following thapsigargin-induced depletion of calcium from the endoplasmic reticulum (EC50’s ≈ 2 µg/ml). Thus, ZnO nanoparticles interfere the M3 signaling pathway (at least) via disruption of store operated calcium entry.

**Figure 5:**
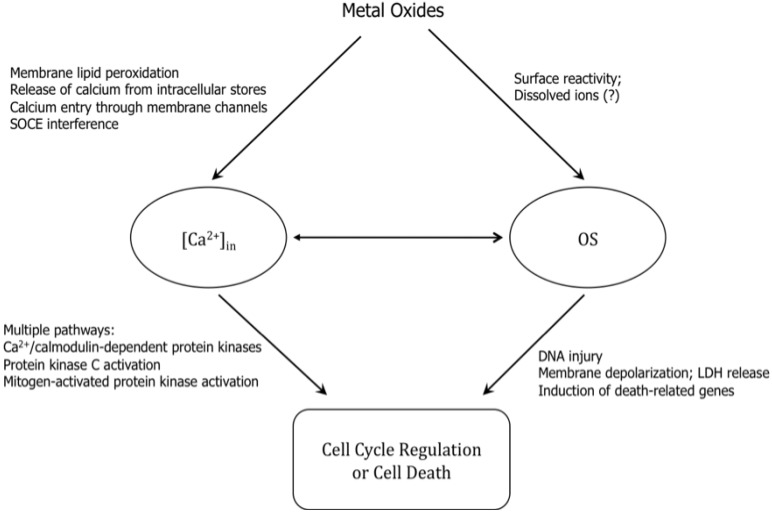
Relationships between ZnO nanoparticles, production of reactive oxygen species (OS), and intracellular Ca^2+^ concentrations. (modified from [43])

Nanoparticle-induced cell death by various means is summarized in **Figure 5** [34,35,37,38,43]. The increases in intracellular content OS may have multiple sources. We postulate that the elevated OS is a consequence of ZnO surface reactivity and/or defects interacting with intracellular reductants in combination with the effects of dissolved metal ions that catalyze redox reactions. Synergistic interactions between intracellular [Ca^2+^] and OS are a likely contributing factor. While [Ca^2+^]_in_ and OS affect the activity of each other, they both induce cell death by distinct pathways. Finally, though calcium-dependent kinase activation pathways leading to cell cycle regulation or cell death have been well documented, studies in this area with transition metal oxides are lacking.

### 6.3. Pro-Inflammatory Response

**Figure 6:**
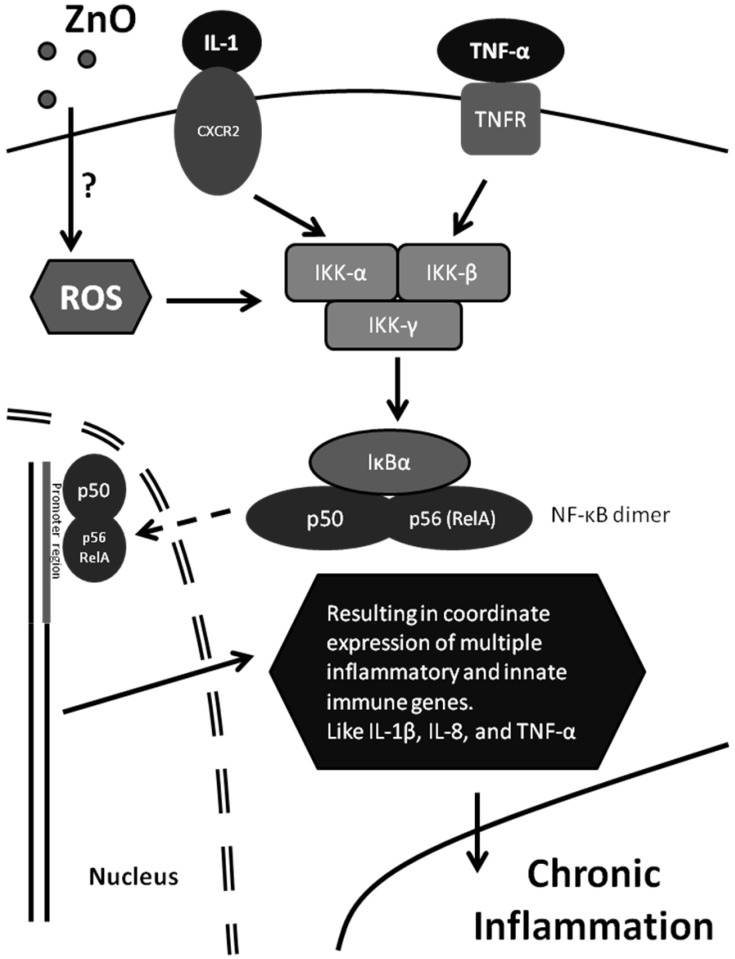
A model shows the simplified NF-kB signaling pathway that is activated by oxidative stress. Persistent activation leads to chronic inflammation.

Inflammation is clinically defined as the presence of redness, swelling and pain. Histologically, it is defined as the presence of edema fluid and the infiltration of tissues by phagocytic cells. Chronic inflammation can lead to diseases such as atherosclerosis, pulmonary diseases, and cancer. Oxidative stress is closely related to inflammatory response by activating the nuclear factor-kappaB (NF-κB) signaling pathway that controls the transcription of pro-inflammatory genes such as IL-1β, IL-8, and tumor necrosis factor-α (TNF-α) (**Figure 6**).

Exposure to nanoparticles has been found to result in oxidative stress-induced activation of pro-inflammatory factors such as IL-1, IL-6, IL-8, and macrophage inflammatory proteins (MIP) at both mRNA and protein levels *in vitro* [45,80,81,82,83,84,85,86]. Additional effects such as activation of IL-2, IL-4, IL-5, IL-6, IL-12, TNF-α, increased number of polymorphonuclear leukocyte (PMN) and lymphocyte, increased B cell distribution, and T cell diminish have been observed *in vivo* (**Table 1**). These factors along with histopathological evidence can be included in the assessment of the risks associated with nanoparticle exposure.

**Table 1:** Pro-inflammatory responses induced by nanoparticles.

Nanoparticle	Size (nm, diameter)	Cell type/animal	Effect	Ref.
TiO_2_	N/A	A549 cells	mRNA and protein of IL-8 ↑	[81]
TiO_2_	20–80	A549 cells	IL-8 mRNA ↑	[80]
TiO_2_	N/A	Human neutrophils	IL-6, IL-8, MIP-1α, MIP-1β ↑	[86]
ZnO	24–70	Lung lavage; BEAS-2B cells	IL-8 mRNA ↑ in both cell types	[82]
Al_2_O_3_, Al	Al_2_O_3_ (33); Al (48)	U937 & A549 (co-cultured)	Phagocytosis activity ↓ (Al); suppress immune response (Al & Al_2_O_3_)	[45]
Au-NPs	50	Bovine retinal pigment epithelial cells	Inhibit VEGF and IL-1β induced proliferation and migration	[83]
Silica	20	HUVEC (human umbilical vein endothelial cells)	IL-6, IL-8, monocyte chemotactic protein-1 α (MIP-1α) ↑	[85]
Fe	20–50	HL1-NB cells (mouse cardiac cells)	IL-8 & MCP-1 not changed	[84]
Fe_3_O_4_	5.3	ICR mouse (♂)	IL-1, IL-2, IL-4, IL-5, IL-6, IL-12, TNF-α, TGF-β, IgE, & B cell distribution ↑. T cell (CD4+/CD8+) diminished.	[87]
Fe_3_O_4_ (superpara magnetic)	36	BALB/c mouse (♂)	PMN & lymphocyte ↑; IL-1β, IL-6, TNF-α, MIP-1α mRNA ↑	[88]
SWCNT; MWCNT	SWCNT: 4(W) x 1E5(L) MWCNT:30(W) x 2E5(L)	BALB/c mouse (♀)	TNF-α & MIP-1 ↑	[89]

## 7. Conclusions

Recent studies have increased our understanding of the nanotoxicity of metal oxide particles, particularly with respect to oxidative stress-induced cascade pathways that lead to inflammatory responses. Several important challenges remain. First, inter-laboratory discrepancies contribute to uncertainties in risk assessment. Second, as new nanomaterials continue to emerge, a systematic approach to identifying the physicochemical properties of nanomaterials that determine toxicity is required. This is problematic using nanomaterials of industrial origin since they 1) vary in multiple physical characteristics, 2) are synthesized using different methods, and 3) vary widely in purity. Third, to attribute toxicity to a particular factor, one needs to consider the extent to which the test design is relevant to the cellular environment. It is likely that toxicity is a non-linear function of multi-variables, which highlights the necessity of developing a theoretical computing model for predictive nanotoxicity. 
